# Pumped double quantum dot with spin-orbit coupling

**DOI:** 10.1186/1556-276X-6-212

**Published:** 2011-03-11

**Authors:** Denis Khomitsky, Eugene Sherman

**Affiliations:** 1 Department of Physics, University of Nizhny Novgorod, 23 Gagarin Avenue, 603950 Nizhny Novgorod, Russian Federation; 2 Department of Physical Chemistry, Universidad del País Vasco, 48080 Bilbao, Spain; 3 IKERBASQUE Basque Foundation for Science, 48011, Bilbao, Spain

## Abstract

We study driven by an external electric field quantum orbital and spin dynamics of electron in a one-dimensional double quantum dot with spin-orbit coupling. Two types of external perturbation are considered: a periodic field at the Zeeman frequency and a single half-period pulse. Spin-orbit coupling leads to a nontrivial evolution in the spin and orbital channels and to a strongly spin- dependent probability density distribution. Both the interdot tunneling and the driven motion contribute into the spin evolution. These results can be important for the design of the spin manipulation schemes in semiconductor nanostructures.

PACS numbers: 73.63.Kv,72.25.Dc,72.25.Pn

## Introduction

Quantum dots, being one of the most intensively studied examples of natural and artificial nanostructures, attract attention due to the richness in the properties they demonstrate in the static and dynamic regimes [1]. A possible realization of qubits for quantum information processing can be done by using spins of electrons in semiconductor quantum dots [2]. Spin- orbit coupling makes the dynamics even in the basic systems such as the single-electron quantum dots extremely rich both in the orbital and spin channels. If the frequency of the electric field driving the orbital motion matches the Zeeman resonance for electron spin in a magnetic field, the spin-orbit coupling causes a spin flip. This effect was proposed in refs. [3,4] to manipulate the spin states by electric means. The efficiency of this process is much greater than that of the conventional application of a periodic resonant magnetic field. The ability to cause coherently the spin flip in GaAs quantum dots was demonstrated in ref. [5] where the gate-produced electric field induced the spin Rabi oscillations. In ref. [6] periodic electric field caused the spin dynamics by inducing electron oscillations in a coordinate-dependent magnetic field. In addition, these results confirmed that the spin dephasing in GaAs quantum dots, arising due to the spin-orbit coupling [7,8] is not sufficiently severe to prohibit a coherent spin manipulation.

The spin dynamics experiments [5,6] necessarily use at least a double quantum dot to detect the driven spin state relative to the spin of the reference electron. Multiple quantum dots realizations become nowadays the subject of extensive investigation [9]. In double quantum dots an interesting charge dynamics occurs and requires theoretical understanding. In this article we address full driven by an external electric field spin and charge quantum dynamics in a one-dimensional double quantum dot [10-13]. Despite the simplicity, these systems show a rich physics. In the wide quantum dots, where the tunneling is suppressed, and the motion is classical, the interdot transfer occurs only due to the over-the-barrier motion, and a chaos-like behavior is usually expected. The irregular driven behavior in the spin and charge dynamics in these systems was studied in ref. [14]. In the quantum double quantum dots, the tunneling between single quantum dots is crucial and the spin-orbit coupling makes the interdot tunneling spin-dependent [15-17]. In quantum systems a finite set of energy eigenstates allows only for a strongly irregular rather than a real chaotic behavior. These orbital and spin dynamical irregularities are important for the understanding of the quantum processes in multiple quantum dots.

In this article we consider various regimes for a one-dimensional double quantum dot with spin-orbit coupling driven by an external electric field and analyze the probability and spin density dynamics in these systems.

## Hamiltonian, time evolution, and observables

**Figure 1:**
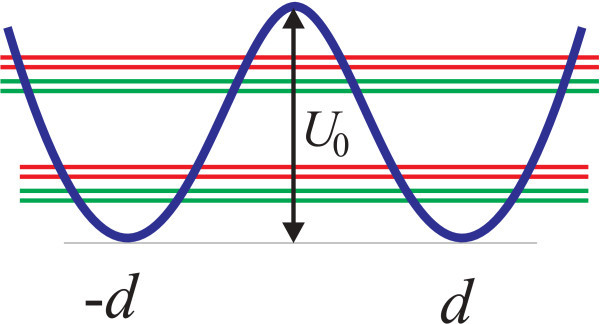
**A schematic plot of the double-well potential described by Equation (1)**. Double green (red) lines correspond to the spin-split even (odd) tunneling-determined orbital states.

We use a quartic potential model to describe a one-dimensional double quantum dot [18],(1)

where the minima located at *d *and -*d *are separated by a barrier of height *U*_0_, as shown in Figure 1. We assume that the interminima tunneling is sufficiently weak such that the ground state can be described with a high accuracy as even linear combination of the oscillator states with a certain "harmonic" frequency *ω*_0 _located near the minima. The double quantum dot is located in a static magnetic field *B_z _*along the *z*-axis and is driven by an external electric field *ℰ*(*t*) parallel to the *x*-axis. The full Hamiltonian , where the time-independent parts are given by(2)(3)

and the time-dependent perturbation is(4)

Here *p_x _*is the momentum operator, *m *is the electron effective mass, *e *is the electron charge, Δ*_z _*= |*g*|*μ_B_B_z _*(we assume below *g *< 0) is the Zeeman splitting, and *σ_i _*are the Pauli matrices. The electron Landé factor *g *determines the effect of *B_z_*, which in this geometry is reduced to the Zeeman spin splitting only. The bulk-originated Dresselhaus (*β*) and structure-related Rashba (*α*) parameters determine the strength of spin-orbit coupling and make the electron velocity defined as(5)

spin-dependent.

We use the highly numerically accurate approach to describe the dynamics with the sum of Hamiltonians in Equations (2)-(4). As the first step we diagonalize exactly the time-independent *H*_0 _+ *H*_so _in the truncated spinor basis *ψ_n_*(*x*) |*σ*〉 of the eigenstates of the quartic potential in magnetic field without spin-orbit coupling with corresponding eigenvalues *E_nσ_*. As a result, we obtain the basis set |*ψ***_n_**〉 where bold **n **incorporates the spin index. For the presentation, it is convenient to introduce the four-states subset: |*ψ*_**1**_〉 = *ψ*_1_(*x*)|↑〉, |*ψ***_2_**〉 = *ψ*_1_(*x*)|↓〉, |*ψ***_3_**〉 = *ψ*_2_(*x*)|↑〉, |*ψ***_4_**〉 = *ψ*_2_(*x*)|↓〉, and to note that the spin-dependent bold index may not correspond to the state energy due to the Zeeman term in the Hamiltonian. The wavefunction *ψ*_1_(*x*) (*ψ*_2_(*x*)) is even (odd) with respect to the inversion of *x*. In the case of weak tunneling, assumed here, these functions can be presented in the form: , where *ψ*_L_(*x*) and *ψ*_R_(*x*) are localized in the left and in the right dot, respectively.

As the second step we build in the full basis the matrix of time-dependent  and study the full dynamics with the wavefunctions:(6)

The expansion coefficients *ξ***_n_**(*t*) are then calculated as:(7)

Where . The spin-dependence of the matrix element of coordinate responsible for the spin dynamics is determined with(8)

and the spin-dependent velocity in Equation (5).

With the knowledge of the time-dependent wavefunctions (6) one can calculate the evolution of probability *ρ*(*x*, *t*) and spin *S_i_*(*x*, *t*)-density(9)(10)

Since we are interested in the interdot transitions, with these distributions we find the gross quantities, e.g., for the right quantum dot:(11)(12)

where *ω*_R_(*t*) is the probability to find electron and  is the analog of expectation value of the spin component.

## Calculations and results

As the electron wavefunction at *t *= 0 we take linear combinations of two out of four low-energy states. The initial state in the form  is localized in the left quantum dot, corresponding to the parameters .

Two types of electric field were considered as the external perturbation. The first one is the exactly periodic perturbation for all *t *> 0:(13)

Where *T_z_*(*B_z_*) = 2π*ħ*/Δ*_z _*is the Zeeman period. The second type is a half-period pulse, same as in Equation (13), but acting at the time interval 0 <*t *<*T_z _*(*B_z_*)/2 only. The spectral width of the pulse covers both the spin and the tunneling splitting of the ground state, thus, driving the spin and orbital dynamics simultaneously. Since *Tz*(*Bz*)*ω *≫ 1, that is the corresponding frequencies are much less than those for the transitions between the orbital levels corresponding to a single dot, the higher-energy states follow the perturbation adiabatically. The field strength *ℰ*_0 _is characterized by parameter *f *such that |*e*|*ℰ*_0 _≡ *f *× *U*_0_/2*d*. Here we concentrate on the regime of a relatively weak coupling (*f *=≫ 1)

where the shape of the quartic potential remains almost intact in time, and the interdot tunneling is still crucially important. For the magnetic field we consider two different regimes Δ*_z _*= Δ*E_g_*/2 and Δ*_z _*= 2Δ*E_g _*to illustrate the role of the Zeeman field for the entire dynamics.

We consider a nanostructure with  nm and *U*_0 _= 10 meV. The four lowest spin-degenerate energy levels are *E*_1 _= 3.938 meV, *E*_2 _= 4.030 meV, E_3 _= 9.782 meV, *E*_4 _= 11.590 meV counted from the bottom of a single quantum dot with the tunneling splitting Δ*E_g _*= *E*_2 _- *E*_1 _= 0.092meV, and the corresponding timescale 2*πħ*/Δ*E_g _*= 45ps. The spin-orbit coupling is described by parameters *α *= 1.0 · 10^-9 ^eVcm and *β *= 0.3 · 10^-9 ^eVcm. The field parameter *f *= 0.125, corresponding to *ℰ*_0 _= 177 V/cm. We use the truncated basis of 20 states with the energies up to 42 meV.

**Figure 2:**
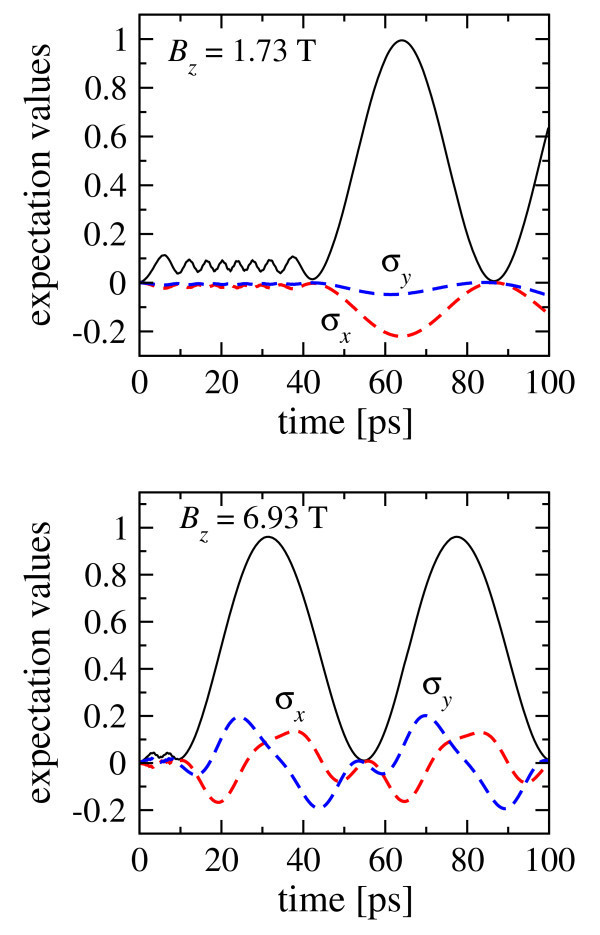
**Motion driven by the exactly periodic field**. Upper panel: *B_z _*= 1.73T, Δ*_z _*= Δ*E_g_*/2 and *T_z _*(*B_z_*) = 90 ps; lower panel: *B_z _*= 6.92T, Δ*_z _*= 2Δ*E_g_*, and *T_z_*(*B_z_*) = 22 ps. The states for *ξ*_n_(*t*) are marked near the plots. The upper panel demonstrates a relatively slow dynamics on the top of the fast oscillations. The increase in the *ξ*_2_(*t*) term corresponds to the possible spin-flip due to the external electric field.

We begin with the exactly periodic driving force, as illustrated in Figure 2 where |*ξ***_n_**|^2^ for three states are presented. Since the motion is periodic, here we use the Floquet method [13,19,20] based on the exact calculation at the first period and then transformed into the integer number of periods. Figure 2 demonstrates the interplay between the tunneling and the spin-flip process. The results indicate that the exact matching of the driving frequency with the Zeeman splitting generates the spin flip which is clearly visible as the initial spin-up (*ξ***_1 _**and *ξ***_3_**) components are decreasing to zero and, at the same time, the opposite spin-down components (*ξ***_2 _**and *ξ***_4_**) reach their maxima (not shown in the upper panel). The spin-flip time is approximately 350*T_z _*(*B_z_*) (or 31 ns) for the weak magnetic field (upper panel) and 24*T_z _*(*B_z_*) (or 528 ps) for the strong field (lower panel). Such an increase in the Rabi frequency with increasing magnetic field is consistent with previous theoretical [3,4] and experimental results [5].

**Figure 3:**
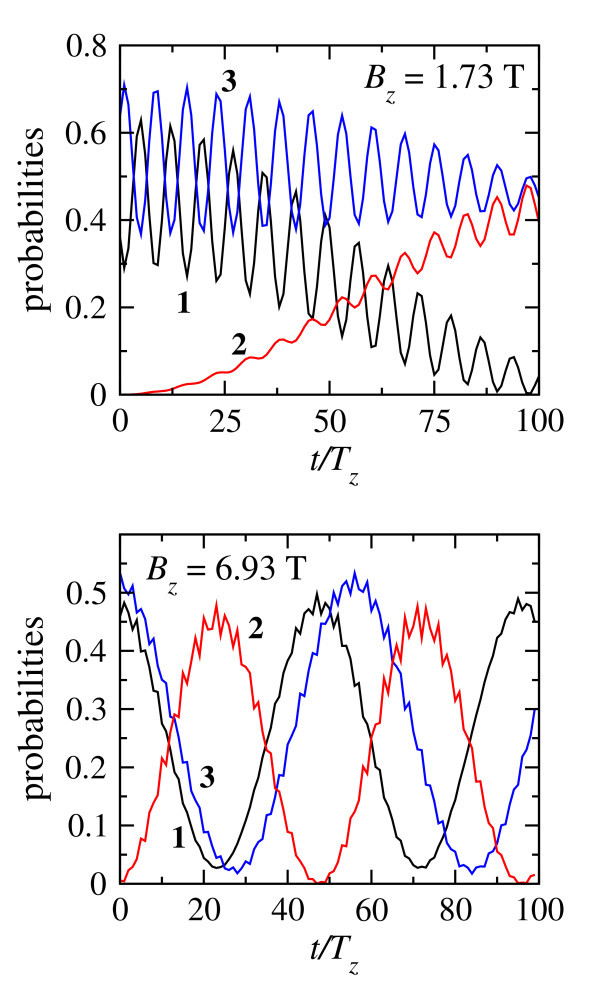
**Motion driven by the single-pulse field**. Upper panel: *B_z _*= 1.73T, Δ*_z _*= Δ*E_g_*/2, and *T_z_*(*B_z_*) = 90 ps; lower panel: *B_z _*= 6.92T, Δ*_z _*= 2Δ*E_g_*, and *T_z_*(*B_z_*) = 22 ps Black line is the probability to find the electron in the right quantum dot. Red and blue dashed lines show corresponding spin components, as marked near the lines, determined both by the spin-orbit coupling and external magnetic field.

As the second example we consider the probabilities *ω*_R_(*t*) and  for the pulse-driven motion, presented in Figure 3. As one can see in the figure, the initial stage is the preparation for the tunneling, which develops only after the pulse is finished. Electric field of the pulse induces the higher-frequency motion by involving higher-energy states, as can be seen in the oscillations at t ≤ *T_z _*(*B_z_*)/2, however, prohibits the tunneling. Such a behavior of the probability and spin density can be explained by taking into account the detailed structure of matrix elements x**_nm_**. Namely, due to the symmetry of the eigenfunctions in a symmetric double QW the largest amplitude can be found for the matrix element of -operator for the pairs of states with opposite space parity having the same dominating spin projection. Hence, the dynamics involving all four lowest levels first of all triggers the transitions inside these pairs which do not involve the spin flip and only after this the spin-flip processes can become significant. As a result, Figure 3 shows that the spin flip has only partial character while the free tunneling dominates as soon as the pulse is switched off. A detailed description of other processes of nonresonant driven dynamics in the case of a half-period perturbation can be found in ref. [21].

## Conclusions

We have studied the full driven quantum spin and charge dynamics of single electron confined in one-dimensional double quantum dot with spin-orbit coupling. Equations of motion have been solved in a finite basis set numerically exactly for a pulsed field and by the Floquet technique for the periodic fields. We explored here the regime of relatively weak coupling to the external field, where a nontrivial dynamics already occurs. Our results are important for the understanding of the effects of spin-orbit coupling for nanostructures as we have demonstrated a possibility to achieve a controllable spin flip at various time scales and in various regimes by the electrical means only.

## Competing interests

The authors declare that they have no competing interests.

## Authors' contributions

DV and ES contributed equally in the development of the model, calculations, interpretation of the results, and preparation of the manuscript. All authors read and approved the final manuscript.
